# A Natural Monoterpene, Carvacrol, Mitigates Bisphenol A-Triggered Hepatorenal Oxidative Damage, Pro-Inflammatory Gene Expression, and Histopathological Alterations in Rats

**DOI:** 10.3390/life16040643

**Published:** 2026-04-10

**Authors:** Nurtaç Küçükbüğrü, Ulas Acaroz

**Affiliations:** 1 Department of Food Hygiene and Technology, Institute of Health Sciences, Afyon Kocatepe University, 03200 Afyonkarahisar, Türkiye; nurtach@hotmail.com; 2 Department of Food Hygiene and Technology, Faculty of Veterinary Medicine, Afyon Kocatepe University, 03200 Afyonkarahisar, Türkiye; 3 Department of Food Hygiene and Technology, Faculty of Veterinary Medicine, Kyrgyz-Turkish Manas University, Bishkek 720038, Kyrgyzstan

**Keywords:** bisphenol A, carvacrol, oxidative stress, inflammation, histopathology, Wistar albino rat

## Abstract

Bisphenol A (BPA) is a widely used endocrine-disrupting chemical that has been linked to oxidative stress and inflammation. This study investigated whether carvacrol (CAR), a natural monoterpene with antioxidant potential, mitigates BPA-induced hepatorenal toxicity in rats. Forty-two male Wistar albino rats were allocated into six groups (*n* = 7/group): control, vehicle (corn oil), BPA (25 mg/kg/day), and BPA co-administered with CAR (12.5, 25, or 50 mg/kg/day) by oral gavage for 30 days. Oxidative status was assessed in liver and kidney homogenates by measuring malondialdehyde (MDA), reduced glutathione (GSH), and the activities of superoxide dismutase (SOD) and catalase (CAT). In addition, histopathological evaluations were performed, and pro-inflammatory gene expression (NF-κB, TNF-α, and IFN-γ) was quantified by RT-qPCR. BPA induced a consistent pro-oxidant pattern, including increased hepatic MDA with depleted antioxidant defenses, and upregulated inflammatory transcripts. Carvacrol attenuated these alterations in a dose-dependent manner, and the CAR50 group was associated with statistically supported improvements across the oxidative stress panel, pro-inflammatory transcript expression, and histopathology scores. Overall, these findings identify carvacrol as a candidate for further preclinical evaluation against BPA-triggered oxidative and inflammatory disturbances in vivo; however, human-relevant extrapolation will require careful attention to dose scaling, bioavailability, and metabolism.

## 1. Introduction

Bisphenol A (BPA) has been addressed at the public health risk level in the recent literature [1] and remains under active regulatory evaluation in the context of foodstuffs [2]. Evidence syntheses also document BPA in multiple exposure-relevant matrices. For example, Careghini et al. [3] reviewed the presence of BPA together with nonylphenols, benzophenones, and benzotriazoles across soils, groundwater, surface water, sediments, and food. In the wastewater context, Česen et al. [4] reported the occurrence of bisphenol compounds and performed source identification. Human exposure has also been assessed through biomonitoring: Zhang et al. [5] reported urinary BPA concentrations and discussed their implications for human exposure in several Asian countries.

Food-contact migration is one of the exposure contexts repeatedly examined for BPA and related bisphenols. Priovolos and Samanidou [6] presented a recent overview of bisphenol A and its analogs released from food-contact materials into foods and beverages, with particular attention to analytical sample preparation strategies. In a food survey setting, Lin et al. [7] investigated the migration of BPA and its related compounds in canned seafood and presented dietary exposure estimation based on measured levels. Beyond BPA alone, biomonitoring-based work has also been used to describe exposure characteristics and to conduct cumulative risk assessment for BPA and its substitutes [8]. Together, these sources document BPA and related bisphenols within exposure pathways relevant to the general population.

Experimental studies have reported oxidative stress- and inflammation-related outcomes after BPA exposure in animal and cellular models. Bindhumol et al. [9] reported that BPA promotes oxidative stress in the rat liver, primarily through enhanced reactive oxygen species production. In line with this, Moon et al. [10] reported impaired mitochondrial function in the liver at doses below the no observed adverse effect level. In mice, Wang et al. [11] reported that BPA induces apoptosis, oxidative stress, and an inflammatory response in the colon and liver in a mitochondria-dependent manner. In rats, Tang et al. [12] investigated BPA-induced liver damage and reported involvement of oxidative stress and the Keap1–Nrf2 pathway. Low-dose exposure has also been examined in relation to hepatic outcomes: Linillos-Pradillo et al. [13] demonstrated that low-dose BPA exposure causes hepatic injury in lactating Long–Evans rats via oxidative stress, inflammatory responses, and apoptotic pathways, and further reported perinatal effects in female offspring at postnatal day 6. Oxidative-stress endpoints have additionally been assessed in other tissues, such as the rat brain [14]. Reviews and mechanistic discussions further extend the focus to broader biological contexts, including reproductive dysfunction [15], alterations in cellular organelles [16], and Wnt signaling [17].

Inflammation and immune-response markers have also been examined in relation to bisphenol exposure. Peinado et al. [18] systematically examined existing human studies to assess the link between exposure to bisphenols, parabens, and benzophenones and inflammation-related outcomes. Liu et al. [19] performed a systematic review and meta-analysis on associations between endocrine-disrupting chemicals and markers of inflammation and immune responses. At the cellular level, Citarella et al. [20] reported BPA-mediated activation of nuclear factor-κB signaling together with increased motility in non-transformed breast cells. In innate immune research, Dallio et al. [21] reported that environmental BPA exposure triggers trained immunity-related pathways in monocytes. A recent review also directly discusses whether bisphenols can alter the inflammation process [22]. In vivo studies combining oxidative stress endpoints with inflammatory gene expression have been reported as well; for example, Acaroz et al. [23] demonstrated that BPA triggers oxidative imbalance, inflammatory gene activation, and metabolic and tissue-level changes in male Wistar albino rats, and further showed that boron exerts a protective effect against these alterations.

In parallel with BPA-focused work, toxicological and mechanistic research increasingly addresses BPA substitutes and analogs. Palacios-Valladares et al. [24] reviewed BPA and its emergent substitutes with emphasis on oxidative stress and its role in vascular dysfunction. Easson et al. [25] examined oxidative stress and endothelial dysfunction as a mechanism linking bisphenol S exposure to vascular disease in human umbilical vein endothelial cells and a mouse model of postnatal exposure. For hazard characterization, Sendra et al. [26] evaluated adverse (geno)toxic effects of BPA and its analogues in a hepatic 3D cell model. A broader critical review has also been published to identify data gaps and improve risk assessment of bisphenol A alternatives for human health [27]. At the literature-structure level, Ni et al. [28] mapped BPA toxicity research through bibliometric analysis. These works collectively illustrate continued investigation of bisphenols across exposure settings, biological mechanisms, and health-relevant endpoints.

**Figure 1:**
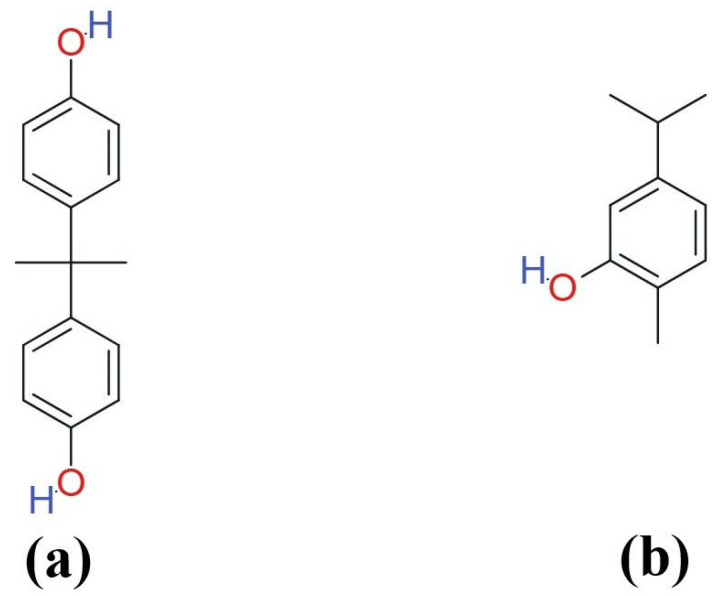
Chemical structures of (**a**) bisphenol A (BPA; 2,2-bis(4-hydroxyphenyl)propane; C_15_H_16_O_2_; MW 228.29 g/mol) and (**b**) carvacrol (5-isopropyl-2-methylphenol; C_10_H_14_O; MW 150.22 g mol^−1^). Both compounds share a phenolic hydroxyl group. BPA is a bisphenolic compound in which two para-hydroxyphenyl rings are connected through an isopropylidene bridge, whereas carvacrol is a monocyclic phenolic monoterpenoid with an isopropyl substituent at C-5 and a methyl group at C-2, making it a positional isomer of thymol.

Carvacrol (CAR; 5-isopropyl-2-methylphenol, C_10_H_14_O) is a naturally occurring phenolic monoterpenoid (a cymene derivative) present in the essential oils of many aromatic plants, particularly Lamiaceae species such as oregano (*Origanum* spp.) and thyme (*Thymus* spp.) [29,30]. Structurally, it is a positional isomer of thymol and contains a substituted phenolic ring that contributes to its lipophilicity and bioactivity [30]. Because essential oils are complex mixtures, the relative abundance of carvacrol varies with plant species, plant part, and chemotype; carvacrol-rich oregano oils can contain carvacrol as the dominant constituent (e.g., approximately 75.7% in *Origanum compactum* essential oil), typically accompanied by related monoterpenes such as thymol, p-cymene, and γ-terpinene [29,31]. BPA (2,2-bis(4-hydroxyphenyl)propane; C_15_H_16_O_2_; MW 228.29 g mol^−1^) features two para-hydroxyphenyl rings connected by an isopropylidene bridge, imparting its estrogenic activity, while carvacrol’s monocyclic phenolic structure is the basis for its radical-scavenging capacity. The chemical structures of both compounds are illustrated in Figure 1.

Carvacrol has been widely discussed as a bioactive monoterpene across multiple health-related contexts. Suntres et al. [32] reviewed the bioactivity and toxicological actions of carvacrol, and Sharifi-Rad et al. [33] provided a comprehensive review of carvacrol and human health. The antioxidant actions of carvacrol have been examined alongside other phenolic compounds [34], and its antimicrobial properties have also been reviewed [30]. Additional syntheses have focused on antioxidant properties relevant to food storage [35], anti-inflammatory and antioxidant activity in the respiratory system [36], and broader anti-inflammatory/antioxidant contexts [37]. Reviews have also discussed the protective effects of carvacrol in relation to lipid profiles, oxidative stress, hypertension, and cardiac dysfunction [38], and neuroprotective relevance [39]. In experimental toxicology models with oxidative stress and inflammation outcomes, Nafees et al. [40] demonstrated that carvacrol alleviates thioacetamide-induced liver toxicity in Wistar rats by reducing oxidative stress, suppressing inflammatory responses, and limiting apoptotic signaling, and El-Sayed et al. [41] showed that thymol and carvacrol reduce doxorubicin-induced cardiotoxic effects in rats by modulating oxidative stress, inflammatory signaling, and apoptosis-related processes. More recently, Gencer et al. [42] described a protective effect of carvacrol against sodium arsenite–induced liver toxicity in rats and explored the involvement of Nrf2/HO-1, RAGE/NLRP3, Bax/Bcl-2/Caspase-3, and Beclin-1–related signaling pathways.

Based on evidence linking BPA exposure to oxidative stress and inflammatory outcomes in experimental models [9,11,12,13,23] and the reported antioxidant/anti-inflammatory properties of carvacrol across toxicant-induced injury settings [32,33,36,40,41,42]. The present study aimed to evaluate whether carvacrol co-administration attenuates BPA-induced hepatorenal damage. The liver and kidney were chosen as key organs of BPA disposition because oral BPA is metabolized mainly in the liver and eliminated via the kidneys. Previous rat studies have also reported oxidative injury in both tissues following BPA exposure [9,23,43]. Therefore, the present study evaluated a 30-day oral exposure model in male Wistar albino rats to (i) quantify BPA-associated alterations in lipid peroxidation and antioxidant defenses (MDA, GSH, SOD, CAT) in liver and kidney, (ii) assess transcriptional activation of key pro-inflammatory mediators (NF-κB, TNF-α, IFN-γ), and (iii) integrate biochemical and molecular findings with semi-quantitative histopathology across a carvacrol dose range (12.5–50 mg/kg/day).

## 2. Materials and Methods

### 2.1. Materials

BPA was purchased from Alfa Aesar (Karlsruhe, Germany) and carvacrol from Sigma-Aldrich Co. (St. Louis, MO, USA). All other reagents and chemicals were of analytical grade and sourced from various commercial suppliers.

### 2.2. Animals and Experimental Protocol

The local committee of Afyon Kocatepe University approved all experimental protocols (Approval No: 49533702–67). The study was conducted in accordance with institutional ethical guidelines and is reported in compliance with the ARRIVE guidelines [44]. Further, 42 Wistar Albino male rats weighing between 200 and 300 g were used in the study. Before starting the experimental phase, the rats were allowed to adapt to the environment for seven days. Animals in the experimental unit were housed in cages under controlled conditions of 24 ± 1 °C with a 12 h light/dark cycle. The environment was regularly ventilated, and the animals were checked daily, receiving fresh water and a standard rat diet.

The carvacrol doses were selected based on published in vivo studies [40,45]. BPA was administered at 25 mg/kg/day by oral gavage, a dose level previously used to induce oxidative damage in rats [43] and comparable to the highest dose (20 mg/kg/day for 30 days) used by Bindhumol et al. [9]. We applied a once-daily schedule for 30 days to generate a reproducible subacute hepatorenal oxidative/inflammatory injury model for testing the mitigation potential of carvacrol; this regimen is not intended to mimic typical environmental human exposure levels.

Wistar Albino male rats were randomly allocated into six groups (n = 7/group) using a simple randomization approach to minimize allocation bias (Table 1). The first group (control group) received 1 mL of physiological saline, while the second group received 1 mL of corn oil. The third group was administered with 25 mg/kg BPA, whereas the fourth group received 25 mg/kg/day BPA along with 12.5 mg/kg/day carvacrol dissolved in corn oil. In the fifth group, 25 mg/kg/day BPA and 25 mg/kg/day carvacrol were administered, while the sixth group received 25 mg/kg/day BPA and 50 mg/kg/day carvacrol, both dissolved in corn oil. All treatments were administered daily via gastric gavage for 30 days.

Two control conditions were intentionally retained. The physiological saline group served as a basal reference for daily oral gavage without a lipid vehicle, whereas the corn oil group served as the treatment-matched vehicle control because BPA and carvacrol were administered in corn oil. For this reason, treatment-related interpretation in this study was based primarily on comparison with the corn oil group, while the saline control was preserved to document baseline physiology. This design also allowed us to directly verify whether corn oil itself influenced the measured endpoints; as shown in the Results, the two control groups remained closely comparable across oxidative, inflammatory, and histopathological outcomes under our experimental conditions. Oil-based vehicle controls are also commonly used in oral BPA rat studies [23,43,46].

### 2.3. Tissue Preparation

At the end of the exposure period, rats were anesthetized with 4% isoflurane administered via inhalation in 30% oxygen. Adequate anesthetic depth was confirmed by monitoring pedal withdrawal reflex and muscle tone. Under deep anesthesia, kidney and liver tissues were collected, and euthanasia was subsequently performed by cervical dislocation in accordance with institutional ethical approval. The collected tissues were promptly processed in the laboratory. Histopathological, biochemical, and molecular analyses were performed on the collected tissues. The preparation of tissue samples was carried out as previously described [47]. Briefly, the kidney and liver tissues of the sacrificed animals were carefully removed and washed with cold isotonic saline buffer. After clearing external tissues, the samples were washed again in chilled Tris-HCl buffer (0.15 M, pH 7.4) and homogenized to prepare a 10% (*w*/*v*) solution. The homogenates were then centrifuged at 2000× *g* for 10 min at 4 °C and stored at −20 °C until analysis. Tissue homogenates were analyzed for lipid peroxidation and antioxidant parameters.

### 2.4. Determination of Oxidative Stress Parameters

Oxidative stress parameters were analyzed in the clarified supernatants of liver and kidney homogenates (10%, *w*/*v*; Section 2.3). Measurements were performed at room temperature using a UV–Vis spectrophotometer (Shimadzu 1601, Tokyo, Japan) with 1 cm path-length cuvettes, and each assay run included a reagent blank. Antioxidant enzyme activities were reported after normalization to tissue protein concentration measured in the same supernatants.

Malondialdehyde (MDA), an index of lipid peroxidation, was measured with the thiobarbituric acid reactive substances (TBARS) assay following Ohkawa et al. [48]. For each sample, tissue supernatant (0.3 mL) was combined with SDS (0.2 mL, 8.1%), followed by acetic acid (1.5 mL, 20%; pH adjusted to 4.0 with NaOH) and thiobarbituric acid (1.5 mL, 0.8%). Distilled water was added to reach a final volume of 4.0 mL. Tubes were heated at 95 °C for 60 min, cooled, and absorbance was recorded at 532 nm. MDA equivalents were calculated from a calibration curve prepared with 1,1,3,3-tetramethoxypropane and expressed as nmol/g wet tissue.

Reduced glutathione (GSH) was measured by the DTNB reaction as described by Beutler et al. [49]. Briefly, 0.2 mL of supernatant was diluted with 1.8 mL of distilled water and deproteinized by adding 3.0 mL of precipitating solution (1.67 g metaphosphoric acid, 0.2 g EDTA, and 30 g NaCl in 100 mL distilled water). After 5 min on ice, samples were filtered (Whatman No. 42) to obtain a clear filtrate. Two milliliters of filtrate were mixed with 8 mL phosphate solution (0.3 M Na_2_HPO_4_) and 1 mL DTNB reagent, and absorbance was recorded at 412 nm. GSH concentrations were obtained from a GSH standard curve and expressed as nmol/g wet tissue.

Superoxide dismutase (SOD) activity was assayed according to Sun et al. [50] based on inhibition of nitroblue tetrazolium (NBT) reduction. In this method, superoxide radicals generated by the xanthine/xanthine oxidase system reduce NBT to a blue formazan dye; SOD in the sample decreases formazan formation by competing for superoxide. The reaction was initiated by xanthine oxidase, incubated at 25 °C, and the absorbance was measured at 560 nm. Percent inhibition was calculated relative to the blank, and one unit (U) of SOD was defined as the amount of enzyme producing 50% inhibition under the assay conditions. The results were expressed as U/µg protein.

Catalase (CAT) activity was determined by monitoring the decomposition of hydrogen peroxide (H_2_O_2_) at 240 nm [51]. The assay (final volume 3.0 mL) contained 50 mM phosphate buffer (pH 7.0) and H_2_O_2_ (10 mM), and the reaction was started by adding the tissue supernatant. The decrease in absorbance was recorded over 45 s, and CAT activity was calculated as the first-order rate constant (k = 2.3/Δt × log(A1/A2)). Activities were normalized to protein content and expressed as k/µg protein (k; nmol min^−1^).

Protein concentration was quantified in the same supernatants using the Lowry (Folin phenol) method [52]. In brief, samples were incubated with freshly prepared alkaline copper reagent for 10 min, followed by rapid addition of diluted Folin–Ciocalteu reagent; absorbance was read at 750 nm after color development. Bovine serum albumin was used to construct the standard curve.

### 2.5. Histopathological Evaluation

Liver and kidney tissues were fixed in 10% buffered formalin. Samples were trimmed to 2–3 mm, placed in cassettes, and washed under running water for 12 h. They were then dehydrated in a graded ethanol series (50–96%), cleared with xylene, and embedded in paraffin. 5–6 µm sections were cut using a microtome (Leica RM 2245, Leica Biosystems, Nussloch, Germany), transferred through a water bath (Leica HI 1210, Leica Biosystems, Nussloch, Germany), and dried at 58 °C for 10 min. Sections were deparaffinized, rehydrated, and stained with the hematoxylin-eosin (H&E). Histopathological analysis was performed using a Nikon Eclipse Ci microscope (Nikon, Tokyo, Japan), and images were captured with a Nikon DS Fi3 digital camera system (Nikon, Tokyo, Japan).

Histopathological lesions were evaluated semi-quantitatively by a blinded veterinary pathologist. Tissue alterations were graded according to the percentage of affected tissue: score 0 (no lesion), score 1 (mild damage, <25% of the tissue affected), score 2 (moderate damage, 25–60%), and score 3 (severe damage, >60%), adapted from previously described histopathological grading approaches [53]. The obtained scores were subsequently used for statistical analysis. For each animal, two sections per organ were prepared and evaluated. The entire tissue section was systematically screened at 20× magnification using a sequential scanning approach, and representative fields showing the most characteristic lesions were selected for photographic documentation.

### 2.6. RT-qPCR Analysis of Inflammatory Gene Expression

Inflammatory gene expression in kidney and liver tissues was evaluated by targeting cytokine-related transcripts (TNF-α and IFN-γ) and an upstream inflammatory signaling mediator (Rela; NF-κB p65 subunit). RNA was isolated from rat tissues stored in RNAlater at −80 °C using the GeneJet RNA Purification Kit(Thermo Fisher Scientific, Waltham, MA, USA). RNA quality and quantity were assessed at A260/A280 using the MultiskanTM FC Microplate Photometer (Thermo Fisher Scientific, Waltham, MA, USA). DNase I treatment was applied, and cDNA synthesis was performed using the RevertAid H Minus cDNA Synthesis Kit. Primers designed based on Rattus norvegicus sequences (NCBI) using FastPCR 6.0. [54] (Table S1). Primer amplification efficiencies were determined using Bio-Rad CFX Manager software, version 3.1 (Bio-Rad Laboratories, Hercules, CA, USA) with all primers showing efficiencies between 95% and 100%. Specificity was further confirmed by melt-curve analysis, showing a single amplification product for each target gene. Gene expression was analyzed via real-time PCR (Bio-Rad, Hercules, CA, USA) with SybrGreen mix. All reactions were performed in triplicate, and no-template controls (NTC) and no-reverse transcription (no-RT) controls were included to exclude contamination and genomic DNA amplification. Relative mRNA expression was calculated using the 2^−ΔΔCt^ method [55] with β-actin as the reference gene for normalization. Results are presented as fold change relative to the control group (set to 1).

### 2.7. Statistical Analysis

Statistical analyses were performed in R (version 4.5.1). Data are presented as mean ± SD (n = 7/group). Normality of model residuals was assessed using the Shapiro–Wilk test. For continuous outcomes (oxidative stress parameters and RT-qPCR fold-change data), between-group differences were evaluated using one-way ANOVA followed by Duncan post hoc test for multiple comparisons. Results are reported as F(df_1_, df_2_), *p*-value, together with effect sizes (η^2^ and ω^2^). Semi-quantitative histopathological scores are presented as median (interquartile range) and were analyzed using the Kruskal–Wallis test followed by Dunn’s post hoc test with Bonferroni correction. A *p*-value < 0.05 was considered statistically significant. For descriptive integration of the multi-parameter response, heatmaps were generated from group mean values after Z-score standardization within each biomarker or gene. In these heatmaps, rows represent individual parameters and columns represent experimental groups; warm colors indicate relatively higher values and cool colors indicate relatively lower values relative to the across-group mean for that specific parameter. The heatmaps were used as visual summaries of coordinated patterns and were not interpreted as separate inferential analyses.

## 3. Results

### 3.1. Oxidative Stress and Antioxidant Defense (MDA, GSH, SOD, CAT)

**Figure 2:**
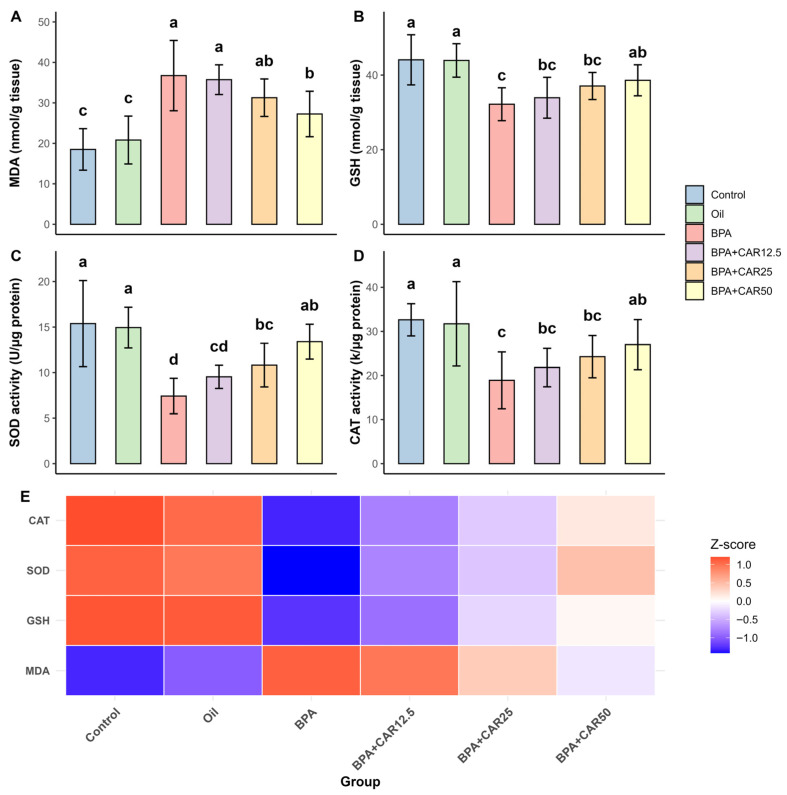
Hepatic oxidative stress parameters. Effects of BPA (25 mg/kg/day) and carvacrol (12.5–50 mg/kg/day) on liver MDA (**A**), GSH (**B**), SOD (**C**), and CAT (**D**) levels after 30 days. Values are mean ± SD (n = 7). Different lowercase letters (a–d) above the bars indicate statistically significant differences among groups based on Duncan’s multiple range test (*p* < 0.001). (**E**) Heatmap shows Z-score-normalized oxidative stress profiles across groups. In panel (**E**), rows correspond to MDA, GSH, SOD, and CAT, while columns correspond to experimental groups; the color scale reflects the relative deviation of each group mean from the across-group mean for that specific marker (warm = higher, cool = lower).

**Figure 3:**
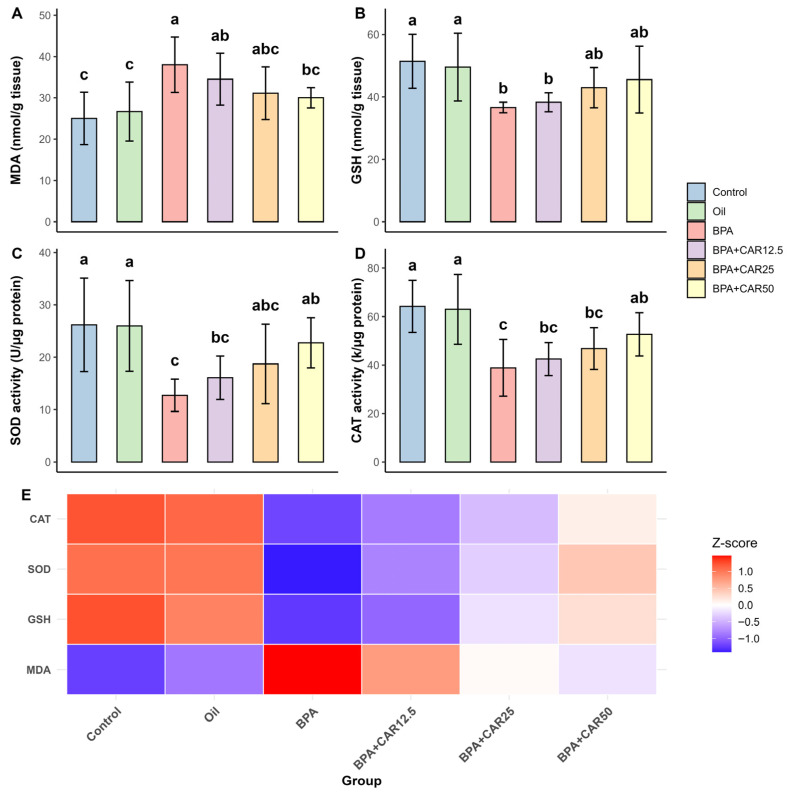
Renal oxidative stress parameters. Effects of BPA (25 mg/kg/day) and carvacrol (12.5–50 mg/kg/day) on kidney MDA (**A**), GSH (**B**), SOD (**C**), and CAT (**D**) levels after 30 days of treatment. Values are expressed as mean ± SD (n = 7). Bars marked with different lowercase letters (a–c) indicate statistically significant differences among groups according to Duncan’s multiple range test. Overall group differences were statistically significant (*p* < 0.01). (**E**) Heatmap showing Z-score–normalized oxidative stress profiles across experimental groups. In panel (**E**), rows correspond to MDA, GSH, SOD, and CAT, while columns correspond to experimental groups; the color scale reflects the relative deviation of each group mean from the across-group mean for that specific marker (warm = higher, cool = lower).

Given that BPA toxicity in the liver–kidney axis is closely associated with oxidative stress, we first quantified lipid peroxidation together with the major antioxidant defense components in these tissues. Malondialdehyde (MDA) was selected as a widely used index of membrane lipid peroxidation [48], whereas reduced glutathione (GSH) reflects the primary non-enzymatic thiol redox buffer [49]; superoxide dismutase (SOD) and catalase (CAT) represent key enzymatic defenses that detoxify superoxide radicals and hydrogen peroxide, respectively [50,51]. This panel has been repeatedly applied to characterize BPA-induced oxidative imbalance in rats [9,23,43]. Across all parameters, the oil group remained comparable to control, indicating that the vehicle did not measurably affect oxidative balance under these conditions (Figure 2 and Figure 3).

BPA exposure shifted hepatic redox status toward oxidation, with higher MDA together with lower GSH, SOD, and CAT (Figure 2). Overall group effects for all hepatic oxidative stress parameters were statistically significant (*p* < 0.001). Hepatic MDA showed a significant treatment effect (F(5, 36) = 11.926, *p* < 0.001; η^2^ = 0.624; ω^2^ = 0.565) and was significantly higher after BPA administration (36.74 ± 8.68) than in control (18.50 ± 5.13) and oil (20.82 ± 5.91). Co-administration of carvacrol reduced MDA in a dose-related manner: CAR12.5 did not differ from BPA (35.73 ± 3.67), CAR25 showed a lower mean value (31.28 ± 4.63) without statistical separation from BPA, and CAR50 significantly decreased MDA (27.25 ± 5.61) compared with BPA, although values remained above the control/oil range (Figure 2A).

Hepatic GSH showed a significant treatment effect (F(5, 36) = 7.130, *p* < 0.001; η^2^ = 0.498; ω^2^ = 0.422). GSH was significantly decreased in the BPA group (32.19 ± 4.41) compared with control (44.09 ± 6.72) and oil (43.92 ± 4.48) (Figure 2B). Carvacrol increased GSH relative to BPA in a dose-related pattern; statistical separation from BPA was observed at CAR50 (38.60 ± 4.16), whereas CAR12.5 (33.92 ± 5.48) and CAR25 (37.07 ± 3.63) remained between BPA and control/oil values (Figure 2B).

Hepatic SOD activity was also significantly reduced by BPA, declining from 15.38 ± 4.73 (control) and 14.94 ± 2.23 (oil) to 7.42 ± 1.95 (BPA) (Figure 2C). Hepatic SOD activity showed a significant treatment effect (F(5, 36) = 10.050, *p* < 0.001; η^2^ = 0.583; ω^2^ = 0.519). Carvacrol co-administration increased hepatic SOD activity compared with BPA. CAR12.5 remained statistically similar to BPA (9.54 ± 1.28), whereas higher doses showed higher SOD activities (CAR25: 10.82 ± 2.39; CAR50: 13.40 ± 1.91), with CAR50 shifting SOD toward control/oil values (Figure 2C).

Similarly, Hepatic CAT activity showed a significant treatment effect (F(5, 36) = 5.653, *p* < 0.001; η^2^ = 0.440; ω^2^ = 0.356). Hepatic CAT activity was significantly lower after BPA exposure (18.90 ± 6.45) than in control (32.63 ± 3.65) and oil (31.72 ± 9.57) (Figure 2D). Carvacrol increased CAT activity in a dose-related manner; CAR50 (26.99 ± 5.69) was significantly higher than BPA, whereas CAR12.5 (21.80 ± 4.36) and CAR25 (24.27 ± 4.80) showed intermediate values (Figure 2D). The liver heatmap summarizes this coordinated response, showing that increasing carvacrol dose progressively shifted the overall hepatic oxidative profile toward control/oil conditions (Figure 2E). In this context, the oxidative heatmaps in Figure 2E and Figure 3E should be read row-by-row: higher MDA appears as a warmer signal in the BPA group, whereas depleted antioxidant defenses (GSH, SOD, and CAT) appear as cooler signals; with carvacrol co-treatment, this pattern progressively shifts back toward the control/oil profile.

BPA exposure also shifted renal oxidative balance toward oxidation, with increased lipid peroxidation and decreased antioxidant defenses, while carvacrol co-treatment attenuated these alterations in a dose-dependent manner (Figure 3). Overall group effects for all renal oxidative stress parameters were statistically significant (*p* < 0.01).

Renal MDA levels were higher in the BPA group (38.02 ± 6.71) than in the control (25.01 ± 6.34) and oil (26.68 ± 7.15) (Figure 3A). Carvacrol co-administration reduced MDA relative to BPA across doses (CAR12.5: 34.52 ± 6.26; CAR25: 31.12 ± 6.38; CAR50: 30.02 ± 2.45), with CAR50 shifting MDA toward the control/oil range (Figure 3A). MDA showed a significant treatment effect (F(5, 36) = 4.422, *p* < 0.01; η^2^ = 0.381; ω^2^ = 0.289).

Renal GSH was lower in the BPA group (36.62 ± 1.68) compared with control (51.43 ± 8.66) and oil (49.56 ± 10.89) (Figure 3B). Carvacrol increased GSH relative to BPA, with dose-related increases at CAR25 (42.97 ± 6.43) and CAR50 (45.55 ± 10.69), and the CAR50 group approached the control/oil range (Figure 3B). GSH showed a significant treatment effect (F(5, 36) = 4.109, *p* < 0.01; η^2^ = 0.363; ω^2^ = 0.270).

Renal SOD activity declined after BPA exposure (12.73 ± 3.08) compared with control (26.19 ± 8.92) and oil (25.98 ± 8.66) (Figure 3C). Carvacrol increased SOD activity relative to BPA (CAR12.5: 16.08 ± 4.14; CAR25: 18.72 ± 7.60; CAR50: 22.77 ± 4.79), with the highest dose shifting values toward control/oil levels (Figure 3C). SOD activity showed a significant treatment effect (F(5, 36) = 4.831, *p* < 0.01; η^2^ = 0.402; ω^2^ = 0.313).

A similar pattern was observed for renal CAT activity. BPA decreased CAT from 64.19 ± 10.76 (control) and 63.00 ± 14.40 (oil) to 38.84 ± 11.68 (BPA) (Figure 3D). Carvacrol increased CAT activity in a dose-dependent manner (CAR12.5: 42.47 ± 6.83; CAR25: 46.81 ± 8.60; CAR50: 52.64 ± 8.92), and the renal heatmap showed a progressive shift in the overall oxidative profile toward control/oil with increasing carvacrol dose (Figure 3D,E). CAT activity showed a significant treatment effect (F(5, 36) = 7.106, *p* < 0.001; η^2^ = 0.497; ω^2^ = 0.421).

The renal heatmap integrates these coordinated changes, illustrating that BPA exposure is associated with a higher MDA signal alongside reduced antioxidant defenses (GSH, SOD, and CAT), whereas increasing doses of carvacrol progressively shift the overall renal oxidative stress profile back toward control/oil conditions (Figure 3E).

### 3.2. Inflammatory Gene Expression in Liver and Kidney

To characterize the inflammatory component of BPA-induced hepatorenal injury and to complement the biochemical oxidative-stress endpoints, we quantified mRNA expression of a focused pro-inflammatory panel comprising NF-κB (a central inflammatory transcriptional regulator) and the cytokine mediators TNF-α and IFN-γ, which have been reported to respond to BPA exposure and/or reflect BPA-triggered inflammatory signaling in experimental models [20,22,23,56]. Accordingly, BPA increased IFN-γ, NF-κB, and TNF-α expression in the liver, whereas carvacrol co-treatment reduced these transcripts in a dose-dependent manner (Figure 3). Overall group effects for all hepatic inflammatory gene expression parameters were statistically significant (*p* < 0.001).

**Figure 4:**
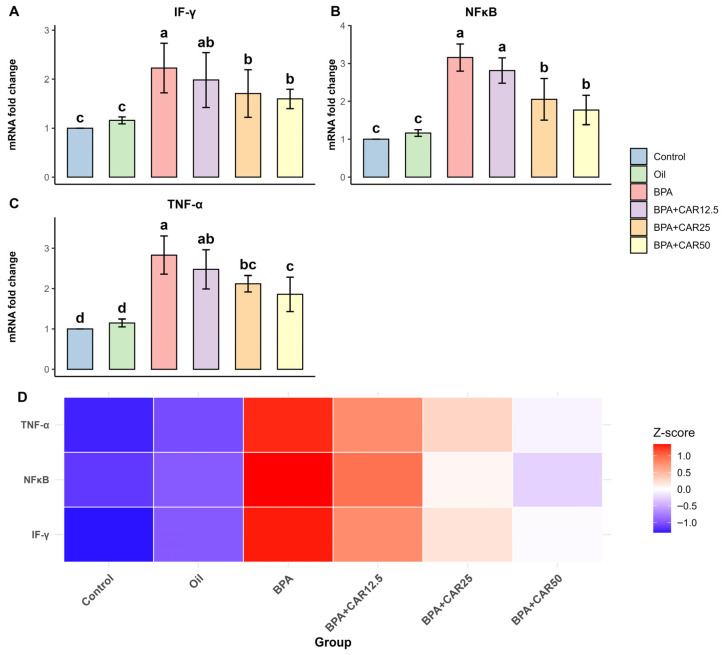
Effects of BPA and carvacrol on hepatic inflammatory gene expression. Hepatic mRNA expression levels of (**A**) IFN-γ, (**B**) NF-κB, and (**C**) TNF-α following BPA (25 mg/kg/day) exposure and carvacrol (12.5–50 mg/kg/day) co-treatment for 30 days. Gene expression levels are presented as fold change relative to the control group. Values are expressed as mean ± SD (n = 7). Bars marked with different lowercase letters (a–d) indicate statistically significant differences among groups according to Duncan’s multiple range test (*p* < 0.001). (**D**) Heatmap illustrating the Z-score–normalized mean expression levels of IFN-γ, NF-κB, and TNF-α across experimental groups, where red indicates higher and blue indicates lower relative expression levels. In panel (**D**), rows correspond to IFN-γ, NF-κB, and TNF-α, while columns correspond to experimental groups; the color scale reflects the relative deviation of each group mean from the across-group mean for that specific transcript (warm = higher/upregulated, cool = lower/downregulated).

BPA exposure elicited a statistically significant pro-inflammatory transcriptional response in liver tissue, with upregulation of IFN-γ, NF-κB, and TNF-α, whereas carvacrol co-treatment downregulated these transcripts in a dose-dependent manner (Figure 4).

BPA significantly upregulated hepatic IFN-γ mRNA expression, increasing from baseline values in the control group (1.00 ± 0.00) and the oil group (1.16 ± 0.07) to 2.23 ± 0.51 in the BPA group (Figure 4A). Carvacrol co-administration downregulated IFN-γ relative to BPA in a dose-related manner. CAR12.5 (1.98 ± 0.56) remained statistically similar to BPA, whereas CAR25 (1.71 ± 0.49) and CAR50 (1.60 ± 0.20) reduced IFN-γ expression and shifted values toward control/oil levels (Figure 4A). Hepatic IFN-γ mRNA expression showed a significant treatment effect (F(5, 36) = 10.935, *p* < 0.001; η^2^ = 0.603; ω^2^ = 0.542).

A similar pattern was observed for hepatic NF-κB expression, which showed a significant treatment effect (F(5, 36) = 45.048, *p* < 0.001; η^2^ = 0.862; ω^2^ = 0.840). BPA significantly upregulated hepatic NF-κB mRNA levels from 1.00 ± 0.00 in control (and 1.16 ± 0.09 in oil) to 3.16 ± 0.36 in the BPA group (Figure 4B). Carvacrol co-administration downregulated NF-κB in a dose-related manner. CAR12.5 (2.81 ± 0.33) showed no statistical separation from BPA, whereas CAR25 (2.05 ± 0.55) and CAR50 (1.77 ± 0.39) reduced NF-κB expression and shifted values toward control/oil levels (Figure 4B).

Hepatic TNF-α expression followed a comparable trend (F(5, 36) = 31.659, *p* < 0.001; η^2^ = 0.815; ω^2^ = 0.785). BPA also upregulated hepatic TNF-α mRNA expression from control (1.00 ± 0.00) and oil (1.15 ± 0.10) levels to 2.83 ± 0.48 in the BPA group (Figure 4C). Carvacrol downregulated TNF-α relative to BPA in a dose-dependent manner (CAR12.5: 2.48 ± 0.49; CAR25: 2.12 ± 0.20; CAR50: 1.86 ± 0.43), and the CAR50 group showed the lowest TNF-α expression among BPA-exposed groups, approaching control/oil values (Figure 4C).

**Figure 5:**
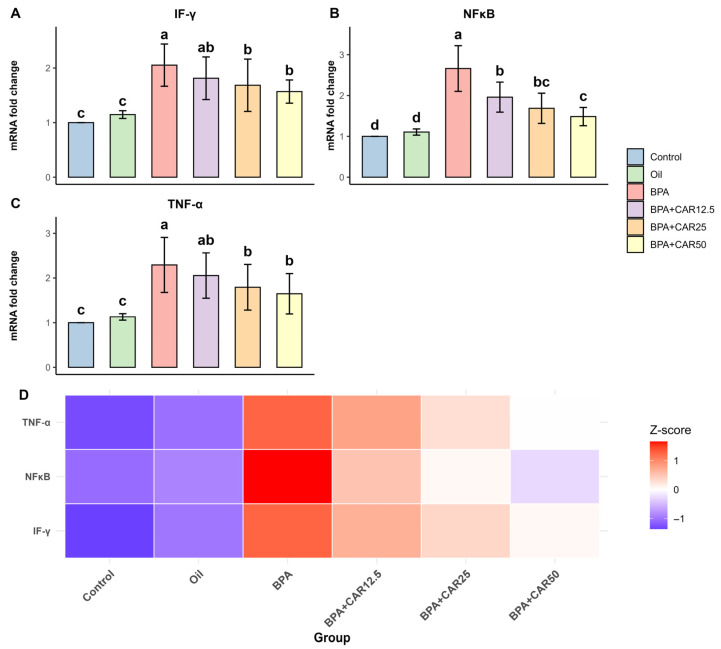
Effects of BPA and carvacrol on renal inflammatory gene expression. Renal mRNA expression levels of (**A**) IFN-γ, (**B**) NF-κB, and (**C**) TNF-α following BPA (25 mg/kg/day) exposure and carvacrol (12.5–50 mg/kg/day) co-treatment for 30 days. Gene expression levels are presented as fold change relative to the control group. Values are mean ± SD (n = 7). Different lowercase letters (a–d) above the bars indicate statistically significant differences among groups according to Duncan’s multiple range test (*p* < 0.001). (**D**) Heatmap showing Z-score–normalized mean expression levels of IFN-γ, NF-κB, and TNF-α across experimental groups, where red indicates relatively higher and blue indicates relatively lower gene expression levels. In panel (**D**), rows correspond to IFN-γ, NF-κB, and TNF-α, while columns correspond to experimental groups; the color scale reflects the relative deviation of each group mean from the across-group mean for that specific transcript (warm = higher/upregulated, cool = lower/downregulated).

The hepatic heatmap integrates these transcriptional changes, demonstrating that BPA exposure is associated with coordinated upregulation of IFN-γ, NF-κB, and TNF-α, whereas increasing doses of carvacrol progressively shift the overall inflammatory gene expression profile toward control/oil conditions (Figure 4D). Likewise, the inflammatory heatmaps in Figure 4D and Figure 5D summarize the coordinated transcriptional response across IFN-γ, NF-κB, and TNF-α, with warmer tones denoting relative upregulation and cooler tones denoting relatively lower expression for each gene.

BPA exposure elicited a statistically significant pro-inflammatory transcriptional response in kidney tissue, with upregulation of IFN-γ, NF-κB, and TNF-α, whereas carvacrol co-treatment downregulated these transcripts in a dose-dependent manner (Figure 4). Overall group effects for all renal inflammatory gene expression parameters were statistically significant (*p* < 0.001).

Renal IFN-γ mRNA expression showed a significant treatment effect (F(5, 36) = 11.631, *p* < 0.001; η^2^ = 0.618; ω^2^ = 0.559). BPA significantly upregulated renal IFN-γ mRNA expression, increasing from baseline levels in the control group (1.00 ± 0.00) and the oil group (1.15 ± 0.07) to 2.05 ± 0.39 in the BPA group (Figure 5A; different superscript groupings). Carvacrol downregulated IFN-γ relative to BPA in a dose-related manner (CAR12.5: 1.81 ± 0.39; CAR25: 1.68 ± 0.48; CAR50: 1.57 ± 0.21), shifting expression toward control/oil levels (Figure 5A).

A similar pattern was observed for renal NF-κB expression, which showed a significant treatment effect (F(5, 36) = 24.511, *p* < 0.001; η^2^ = 0.773; ω^2^ = 0.737). BPA significantly upregulated renal NF-κB mRNA levels from 1.00 ± 0.00 in control (and 1.11 ± 0.08 in oil) to 2.66 ± 0.56 in the BPA group (Figure 5B). Carvacrol co-administration downregulated NF-κB in a dose-related manner (CAR12.5: 1.96 ± 0.37; CAR25: 1.69 ± 0.37; CAR50: 1.48 ± 0.22), and the highest dose produced the lowest NF-κB level among BPA-exposed groups, although values remained above the control/oil baseline (Figure 5B).

Renal TNF-α expression followed a comparable trend (F(5, 36) = 9.796, *p* < 0.001; η^2^ = 0.576; ω^2^ = 0.512). BPA upregulated renal TNF-α mRNA expression from control (1.00 ± 0.00) and oil (1.13 ± 0.07) levels to 2.29 ± 0.62 in the BPA group (Figure 5C). Carvacrol downregulated TNF-α relative to BPA in a dose-dependent manner (CAR12.5: 2.05 ± 0.51; CAR25: 1.79 ± 0.51; CAR50: 1.65 ± 0.45), shifting TNF-α expression toward control/oil values (Figure 5C).

The renal heatmap integrates these transcriptional changes, demonstrating that BPA exposure is associated with coordinated upregulation of IFN-γ, NF-κB, and TNF-α, whereas increasing doses of carvacrol progressively shift the overall renal inflammatory gene expression profile toward control/oil conditions (Figure 5D).

Considering these results, the direct control-versus-CAR50 comparison indicates that the highest carvacrol dose substantially attenuated, but did not completely abolish, the BPA-induced pro-inflammatory transcriptional response. In both liver and kidney, IFN-γ, NF-κB, and TNF-α were clearly reduced relative to BPA, yet they remained above the corresponding control/oil means; across Figure 2, Figure 3, Figure 4 and Figure 5 overall, recovery at CAR50 was therefore best interpreted as endpoint-dependent attenuation rather than full normalization.

### 3.3. Histopathological Findings

**Figure 6:**
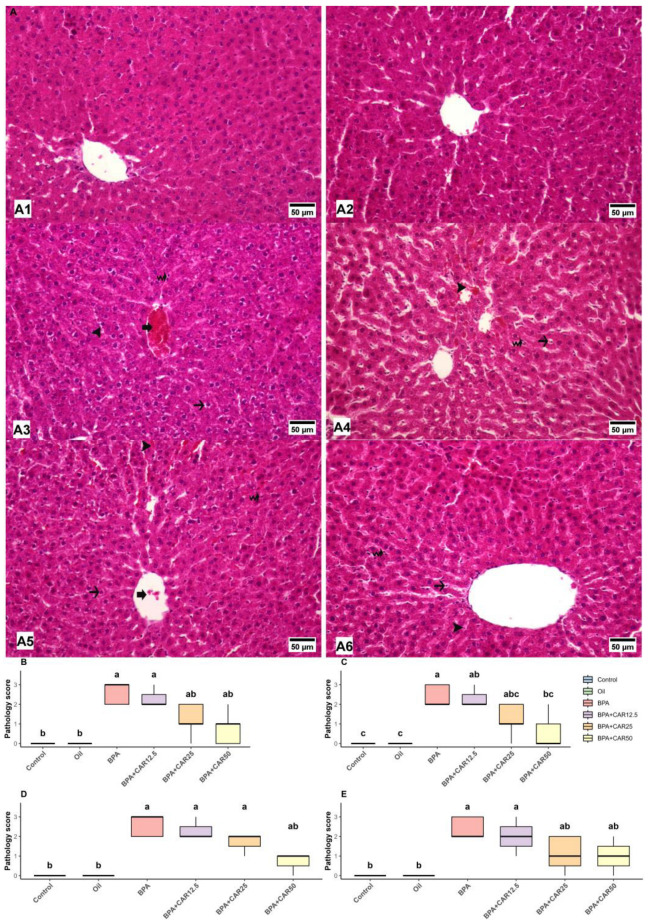
Histopathological alterations and pathology scores in liver tissue. (**A**) Representative H&E-stained liver sections from experimental groups: control (**A1**), oil (**A2**), BPA (**A3**), BPA + CAR12.5 (**A4**), BPA + CAR25 (**A5**), and BPA + CAR50 (**A6**). Hyperemia in the central vein is indicated by thick arrows, while vacuolar degeneration in hepatocytes is marked with thin arrows. An increased number of Kupffer star cells is represented by a curved arrow, and sinusoidal dilatation accompanied by hyperemia is highlighted by arrowheads. Scale bar = 50 µm. (**B**–**E**) Semi-quantitative pathology scores representing central vein hyperemia (**B**), vacuolar degeneration in hepatocytes (**C**), increased Kupffer cell number (**D**), and sinusoidal dilatation with hyperemia (**E**). Data are presented as median with interquartile range (n = 7). Differences among groups were analyzed using the Kruskal–Wallis test followed by Dunn’s multiple comparison test with Bonferroni correction. Different lowercase letters (a–c) indicate statistically significant differences (*p* < 0.05).

**Figure 7:**
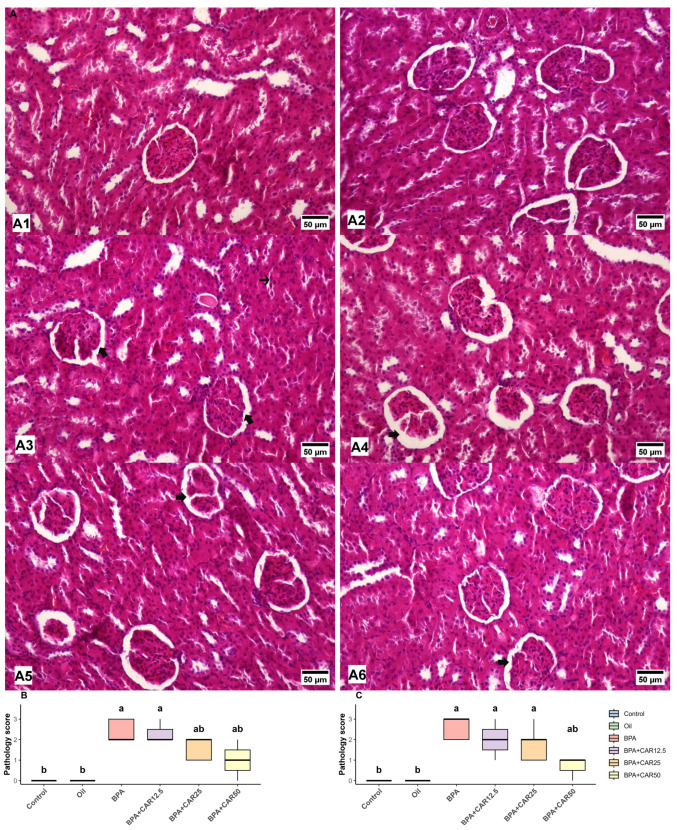
Histopathological alterations and pathology scores in kidney tissue. (**A**) Representative H&E-stained kidney sections from experimental groups: control (**A1**), oil (**A2**), BPA (**A3**), BPA + CAR12.5 (**A4**), BPA + CAR25 (**A5**), and BPA + CAR50 (**A6**). Thick arrows indicate expansion of the glomerular Bowman’s space, whereas thin arrows highlight hyaline cylinder formations within the renal tubules. Scale bar = 50 µm. (**B**,**C**) Semi-quantitative pathology scores representing glomerular Bowman’s space expansion (**B**) and hyaline cylinder formation in renal tubules (**C**). Data are presented as median with interquartile range. Different letters indicate significant differences among groups. Data are presented as median with interquartile range (n = 7). Differences among groups were analyzed using the Kruskal–Wallis test followed by Dunn’s multiple comparison test with Bonferroni correction. Different lowercase letters (a, b) indicate statistically significant differences (*p* < 0.05).

To confirm whether the biochemical and transcriptional changes translated into tissue-level injury, and because BPA exposure has been reported to induce characteristic morphological lesions in the liver and kidney in rats, we performed H&E-based histopathological evaluation with semi-quantitative scoring. Scoring targeted hallmark hepatorenal lesions previously described after BPA exposure, focusing on vascular/parenchymal alterations in the liver and glomerular/tubular alterations in the kidney [43,46,57]. In line with this assessment, BPA exposure increased lesion scores in both tissues, whereas carvacrol co-treatment reduced these scores in a dose-dependent manner (Figure 6 and Figure 7). Overall group effects for all histopathological parameters were statistically significant (*p* < 0.001).

In the control and oil groups, liver architecture was preserved, with no evidence of central vein hyperemia, vacuolar degeneration of hepatocytes, increased Kupffer cell number, or sinusoidal dilatation (all scores: 0 [0–0]; Figure 6(A1,A2)). In contrast, BPA administration resulted in pronounced histopathological injury. Central vein hyperemia scores increased to 3 (2–3), accompanied by marked vacuolar degeneration in hepatocytes [2 (2–3)], a substantial increase in Kupffer cell number [3 (2–3)], and sinusoidal dilatation with hyperemia [2 (2–3)] (Figure 6(A3)).

Carvacrol co-treatment moderated these BPA-induced hepatic lesions. In the BPA + CAR12.5 group, central vein hyperemia and vacuolar degeneration remained elevated [both 2 (2–3)], with persistent increases in Kupffer cell number [2 (2–3)] and sinusoidal dilatation [2 (1–3)], showing limited histological improvement relative to BPA alone (Figure 6(A4)). In contrast, the BPA + CAR25 group exhibited clearer attenuation of hepatic injury, with reduced central vein hyperemia [1 (1–2)] and vacuolar degeneration [1 (1–2)], alongside partial reductions in Kupffer cell number [2 (1–2)] and sinusoidal dilatation [1 (0–2)] (Figure 6(A5)).

The most pronounced histopathological improvement was observed in the BPA + CAR50 group. In this group, central vein hyperemia scores decreased to 1 (0–1), vacuolar degeneration was largely absent [0 (0–1)], Kupffer cell number was reduced to 1 (0–1), and sinusoidal dilatation with hyperemia was attenuated to 1 (0–2), shifting multiple parameters toward control/oil levels by post hoc comparison (Figure 6(A6)).

Semi-quantitative pathology scores corroborated these observations, demonstrating that BPA induced severe hepatic lesions across all evaluated parameters, whereas increasing doses of carvacrol progressively mitigated vascular, cellular, and inflammatory alterations in liver tissue (Figure 6B–E).

Histopathological evaluation of kidney sections demonstrated prominent structural alterations following BPA exposure, whereas carvacrol co-treatment attenuated these lesions in a dose-dependent manner (Figure 7). Overall group effects for all evaluated renal histopathological parameters were statistically significant (*p* < 0.001).

In the control and oil groups, renal architecture was preserved, with normal glomerular and tubular morphology and no evidence of glomerular Bowman’s space expansion or hyaline cylinder formation in renal tubules (both parameters: 0 [0–0]; Figure 6(A1,A2)). In contrast, BPA administration resulted in marked renal injury, characterized by pronounced expansion of the glomerular Bowman’s space [2 (2–3)] and extensive hyaline cylinder formation within renal tubules [3 (2–3)] (Figure 7(A3)).

Carvacrol co-treatment moderated these BPA-induced renal lesions. In the BPA + CAR12.5 group, glomerular Bowman’s space expansion remained elevated [2 (2–3)], and hyaline cylinder formation persisted [2 (1–3)], indicating limited histopathological improvement compared with BPA alone (Figure 7(A4)). In the BPA + CAR25 group, partial attenuation of renal damage was observed, with Bowman’s space expansion reduced to 2 (1–2) and hyaline cylinder formation decreased to 2 (1–2), reflecting an intermediate level of recovery (Figure 7(A5)).

The most pronounced histopathological improvement was evident in the BPA + CAR50 group. In this group, glomerular Bowman’s space expansion declined to 1 (0–2), and hyaline cylinder formation was reduced to 1 (0–1), shifting both parameters toward control/oil levels (Figure 7(A6)).

Semi-quantitative pathology scores supported these observations, demonstrating that BPA induced substantial glomerular and tubular injury in kidney tissue, whereas increasing doses of carvacrol progressively mitigated these structural alterations (Figure 7B,C).

## 4. Discussion

### 4.1. Oxidative Stress and Antioxidant Disruption

In this subacute model, BPA shifted both liver and kidney homogenates toward a pro-oxidant state, as reflected by increased MDA and concomitant reductions in GSH, SOD, and CAT. It should be noted that this pattern was internally consistent across tissues and across complementary endpoints, supporting that lipid peroxidation occurred in the context of a weakened antioxidant defense rather than an isolated change in a single marker. The absence of changes in the vehicle group strengthens the inference that the observed biochemical imbalance was driven by BPA and modified by carvacrol rather than by corn oil per se.

This oxidative fingerprint is well aligned with prior rat studies, where BPA exposure has been linked to hepatic ROS generation and oxidative damage [9] and to hepatorenal oxidative stress accompanied by tissue injury [23,43,46,57]. Our data add to this literature by showing that the same MDA↑/GSH–SOD–CAT↓ pattern can be concurrently detected in the liver–kidney axis under a single exposure regimen, and that it can be dose-dependently attenuated by carvacrol, with the most consistent normalization at 50 mg/kg/day.

Although we did not interrogate upstream redox-regulatory pathways, the coordinated depletion of enzymatic and non-enzymatic antioxidant defenses suggests that BPA exposure either increases ROS production and/or impairs the capacity to detoxify ROS. This interpretation is mechanistically compatible with mitochondrial dysfunction described at lower BPA doses [10] and with pathway-level evidence implicating Keap1–Nrf2 signaling in rat liver injury [12]. Future pathway-focused work will be important to distinguish whether carvacrol primarily acts by limiting ROS generation, enhancing antioxidant gene programs, or both.

### 4.2. Inflammatory Signaling and NF-κB Activation

At the transcriptional level, BPA induced a concordant increase in NF-κB, TNF-α, and IFN-γ in both liver and kidney, indicating that the biochemical oxidative imbalance was accompanied by activation of pro-inflammatory signaling. The fact that carvacrol reduced these transcripts in parallel with the oxidative stress mitigation—again most prominently at 50 mg/kg/day—supports that its protective profile in this model is not limited to antioxidant readouts but extends to inflammation-related molecular responses.

NF-κB is a central node where oxidative signals and inflammatory gene expression intersect; ROS can contribute to NF-κB activation, and NF-κB-driven cytokine signaling can further amplify oxidative damage, creating a self-reinforcing loop. BPA has been shown to activate NF-κB signaling in cell-based systems [20] and inflammatory transcript changes alongside oxidative stress and histopathology have been reported in rat BPA exposure studies [23]. In the kidney, low-dose BPA has also been reported to exacerbate oxidative stress together with elevated inflammatory mediator expression under hypertensive conditions [58], underscoring that renal inflammatory activation is a recurring feature across BPA models.

Although our model used doses above typical environmental exposure and therefore cannot be translated directly to humans, the direction of our inflammatory findings still fits well with the broader literature. Peinado et al. [18] reported in their systematic review that most human studies linked bisphenol exposure—mainly BPA—with higher levels of pro-inflammatory biomarkers, especially C-reactive protein and IL-6. In the same vein, Liu et al. [19] showed in a meta-analysis that BPA exposure was positively associated with circulating CRP and IL-6, supporting the view that endocrine-disrupting chemicals can shift the inflammatory milieu in humans. This interpretation is also supported by mechanistic work in human primary monocytes: Dallio et al. [21] demonstrated that low, environmentally relevant BPA concentrations induced a trained-immunity phenotype, leading to stronger TNF-α and IL-6 responses after LPS restimulation. Collectively, these findings suggest that the NF-κB/TNF-α/IFN-γ upregulation observed in our rats is not an isolated result of this model, but part of a broader pattern in which BPA primes inflammatory signaling across biological systems. Thus, carvacrol appears to mitigate BPA toxicity not only by improving redox status, but also by dampening the associated inflammatory gene response in both tissues.

### 4.3. Histopathological Correlates of Hepatorenal Injury

A key strength of our design is that biochemical and transcriptional shifts were anchored to tissue-level phenotypes. In the liver, BPA exposure produced vascular and parenchymal alterations—central vein hyperemia, hepatocellular vacuolar degeneration, increased Kupffer cell number, and sinusoidal dilatation with hyperemia—consistent with hepatocellular stress and an inflammatory milieu. The graded improvement across the carvacrol dose range, particularly the near-baseline lesion scores in the BPA + CAR50 group, reinforces that biochemical mitigation translated into structural preservation rather than representing an isolated fluctuation in individual biomarkers.

The kidney showed a similarly coherent pattern: BPA increased Bowman’s space expansion and hyaline cylinder formation in renal tubules, findings that align with combined glomerular and tubular injury. Carvacrol reduced these lesions in a dose-related manner, with the highest dose producing the clearest attenuation. The parallel directionality across oxidative, transcript, and histological domains increases confidence that carvacrol moderated a shared injury process rather than acting on a single downstream marker.

Comparable hepatorenal lesions have been documented in BPA-exposed rats in studies. Korkmaz et al. [43] described hepatic necrosis and congestion after BPA exposure. Poormoosavi et al. [46] reported dilated and congested central and portal veins together with inflammatory areas, while Eweda et al. [57] observed central vascular injury, vacuolated hepatocytes, dilated sinusoids, and inflammatory cell accumulation. Acaroz et al. [23] likewise reported sinusoidal dilatation, hyperemia, and degenerative changes in hepatocytes. Our findings reproduce the same general pattern of vascular and degenerative liver injury, and they add semi-quantitative evidence that carvacrol reduced lesion severity stepwise across the dose range.

The kidney findings were equally informative. BPA increased Bowman’s space expansion and hyaline cylinder formation in renal tubules, indicating combined glomerular and tubular injury. These alterations are in line with previous reports of renal tubular casts, tubular epithelial degeneration, and glomerular changes in BPA-exposed rats [23,46]. In our study, the dose-related reduction in these renal lesions by carvacrol is important because it shows that the biochemical and transcriptional improvements were translated into preserved tissue architecture. Thus, the overall meaning of our findings is not just that carvacrol changed biomarkers, but that it limited the morphologic expression of BPA-induced hepatorenal damage.

### 4.4. Carvacrol as a Protective Modulator

Carvacrol’s protective profile in our study is mechanistically consistent given its phenolic structure and documented antioxidant actions, including radical-scavenging activity demonstrated in classic in vitro comparisons with other phenolic compounds [34]. Broader reviews also describe carvacrol as a multifunctional agent with antioxidant and anti-inflammatory potential across biological systems [29,32,33].

Within our BPA model, carvacrol co-administration produced a coherent, dose-dependent shift toward control values across lipid peroxidation, antioxidant defenses, NF-κB-linked inflammatory transcripts, and pathology scores. Notably, the pattern was not all-or-nothing: the lowest dose provided limited correction in several readouts, whereas CAR50 was the most consistently protective across both organs. However, a direct comparison of the control and CAR50 groups indicates strong attenuation rather than complete abolition, particularly for the pro-inflammatory transcripts, which remained above baseline in both liver and kidney. Because a carvacrol-only arm was not included, these data should be interpreted as mitigation within a BPA-challenged background rather than as a formal dissection of carvacrol’s standalone effects on basal oxidative or inflammatory status. Even so, the present design still allows a reliable comparison of BPA versus BPA + CAR groups and therefore supports the conclusion that carvacrol attenuated BPA-related deviations under the conditions tested.

Carvacrol has been evaluated in multiple toxicant-driven injury settings where oxidative stress and inflammation converge, and reviews consistently describe it as a biologically active phenolic monoterpene with antioxidant and anti-inflammatory potential rather than an inert comparator compound [32,33]. Experimentally, it attenuated thioacetamide-induced hepatotoxicity in Wistar rats [40], mitigated cisplatin-induced nephrotoxicity [45], and reduced doxorubicin-induced cardiotoxicity [41]. In a more recent hepatotoxicity model, carvacrol’s protection was discussed in relation to redox and inflammatory pathway modulation, including Nrf2/HO-1 and inflammasome-related signaling [42]. Although we did not directly assess these pathways, our integrated biochemical–transcriptional–histological improvements are consistent with the broader literature positioning carvacrol as a modulator of oxidative–inflammatory networks.

Our findings should therefore be viewed as preliminary experimental evidence that targeting oxidative–inflammatory biology can attenuate BPA-triggered hepatorenal injury in vivo, rather than as direct evidence for efficacy under real-world exposure conditions.

Carvacrol is a dietary monoterpene present in culinary herbs and essential oils, but systemic exposure after oral intake is influenced by absorption, first-pass metabolism, and clearance. Accordingly, the relationship between dietary intake, circulating/tissue levels, and the doses used in animal gavage studies remains a key translational gap. Reviews summarizing its bioactivity and toxicological actions can inform this discussion [32,38], but dedicated pharmacokinetic/ADME studies (including metabolite profiling and tissue distribution) and safety evaluations, alongside lower-dose BPA paradigms, will be needed before any human-relevant inferences can be made.

### 4.5. Strengths and Limitations

Several aspects strengthen confidence in the present findings. First, the outcomes were triangulated across biochemical (MDA, GSH, SOD, CAT), molecular (NF-κB, TNF-α, IFN-γ), and histological endpoints, reducing the likelihood that conclusions are driven by a single assay domain. Second, the dose-range design allowed us to detect a graded protective pattern, which is especially valuable when interpreting natural products that may show threshold-like effects. Third, evaluating both liver and kidney provides a more integrated representation of BPA-associated injury along the metabolism–excretion axis than single-organ approaches.

Limitations are also important for balanced interpretation. Inflammation was assessed at the transcript level without protein-level cytokine quantification; therefore, future studies should corroborate these changes using immunoassays. Likewise, pathway-level redox regulators (e.g., Keap1–Nrf2 signaling) were not measured, limiting mechanistic attribution [12]. Functional clinical chemistry markers (e.g., ALT/AST or BUN/creatinine) were not included, so the present conclusions are centered on tissue oxidative–inflammatory injury rather than organ function. In addition, the study focused on male rats and did not include a carvacrol-only group. Consequently, the extent to which carvacrol alone may shift basal oxidative or inflammatory markers under our experimental conditions cannot be separated from its mitigation of BPA-induced injury. For this reason, our conclusions are intentionally restricted to attenuation within BPA-exposed animals rather than to a complete characterization of carvacrol’s intrinsic baseline effects. Finally, H&E-based pathology provides a comprehensive overview of structural injury but cannot resolve immune cell phenotypes, motivating incorporation of immunohistochemical approaches in future work.

## 5. Conclusions

BPA exposure generated a statistically significant oxidative–inflammatory injury signature in liver and kidney, and carvacrol co-administration shifted this integrated profile toward control/oil across biochemical, transcriptional, and histopathological readouts. Across the dose range tested, attenuation followed a dose-related pattern and CAR50 was associated with the strongest overall protection; however, the highest dose did not completely normalize every endpoint, particularly the pro-inflammatory gene-expression profile, indicating substantial mitigation rather than complete abolition of the BPA-induced phenotype. These findings provide experimental evidence that modulation of oxidative stress and downstream inflammatory signaling can attenuate BPA-associated tissue injury in vivo. Because a carvacrol-only arm was not included, the present findings should be interpreted as evidence of mitigation of BPA-associated alterations rather than as isolation of the standalone effects of carvacrol on basal hepatorenal redox or inflammatory status.

Nevertheless, the present work used a subacute high-dose gavage model and was not designed to reflect typical human exposure conditions. Translational interpretation therefore requires careful consideration of dose scaling and of carvacrol pharmacokinetics, bioavailability, and metabolism. Future studies integrating pharmacokinetic/ADME analyses, functional clinical chemistry endpoints, and environmentally relevant BPA exposures will be necessary to bridge this translational gap. Accordingly, carvacrol should be regarded as a candidate for further preclinical investigation rather than direct evidence for human dietary or therapeutic efficacy.

## Figures and Tables

**Table 1:** Experimental groups and dosing regimen (n = 7 per group).

Groups		BPA (mg/kg/Day)	CAR (mg/kg/Day)	Vehicle
I	Control	-	-	Physiological saline
II	Vehicle	-	-	Corn oil
III	BPA	25	-	Corn oil
IV	BPA + CAR (Low)	25	12.5	Corn oil
V	BPA + CAR (Medium)	25	25	Corn oil
VI	BPA + CAR (High)	25	50	Corn oil

## Data Availability

The data presented in this study are available on reasonable request from the corresponding author.

## Supplementary Materials

The following supporting information can be downloaded at: https://www.mdpi.com/article/10.3390/life16040643/s1, Table S1. Primer sequences used in the study.

## Abbreviations

The following abbreviations are used in this manuscript:

ANOVA	Analysis of variance
BPA	Bisphenol A
CAR	Carvacrol
CAT	Catalase
cDNA	Complementary DNA
Ct	Cycle threshold
DNase	Deoxyribonuclease
DTNB	5,5′-Dithiobis(2-nitrobenzoic acid)
EDTA	Ethylenediaminetetraacetic acid
EFSA	European Food Safety Authority
GSH	Reduced glutathione
H&E	Hematoxylin and eosin
H_2_O_2_	Hydrogen peroxide
IFN-γ	Interferon gamma
LPO	Lipid peroxidation
MDA	Malondialdehyde
mRNA	Messenger RNA
NBT	Nitroblue tetrazolium
NCBI	National Center for Biotechnology Information
NF-κB	Nuclear factor kappa B
PCR	Polymerase chain reaction
RNA	Ribonucleic acid
ROS	Reactive oxygen species
SD	Standard deviation
SYBR	SYBR Green
TBA	Thiobarbituric acid
TNF-α	Tumor necrosis factor alpha
*w*/*v*	Weight/volume
