# Supplementary material for: A Natural Monoterpene, Carvacrol, Mitigates Bisphenol A-Triggered Hepatorenal Oxidative Damage, Pro-Inflammatory Gene Expression, and Histopathological Alterations in Rats
Source: Life (Basel). 2026 Apr 10;16(4):643. doi: 10.3390/life16040643 (PMC13117251; doi:10.3390/life16040643)
Supplement: Supplementary file 1 [file life-16-00643-s001.zip › life-4159569-supplementary.pdf]

Supplementary Materials

A Natural Monoterpene, Carvacrol, Mitigates Bisphenol A–Triggered Hepatorenal Oxidative Damage, Pro-Inflammatory Gene Expression, and Histopathological Alterations in Rats

CONTENTS

Table S1. Primer sequences used in the study

Gene	Primer	Oligonucleotide Sequence (5′–3′)	GenBank Accession No.
β-Actin	F	GAGGGAATCGTGCGTGACAT	NC_005111.4
	R	ACACTGCTGGAAGGTGGACA	
NF-κB	F	TCCCCAAGCCAGCACCCAGC	NM_199267.2
	R	GGCCCCCAAGTCTTCATCAGC	
TNF-α	F	CGAGTGACAAAGCCCGTAGCC	NM_012675.3
	R	GGATGAACACGGCCAGTCGCC	
IFN-γ	F	AAGACAACCGCATCCATCAGCA	NM_138880.3
	R	TTCACCTCGAACTTGGCGATGC	
